# Supplementary material for: Orb2 enables rare-codon-enriched mRNA expression during Drosophila neuron differentiation
Source: Nat Commun. 2024 Jun 20;15:5270. doi: 10.1038/s41467-024-48344-8 (PMC11190236; doi:10.1038/s41467-024-48344-8)

SUPPLEMENTAL DATA

Supplemental Datasets

Supplemental Dataset 1. Sequences of new transgenes presented in this study.

Supplemental Dataset 2. Data for RNAi screen.

Supplemental Dataset 3. RNAseq data comparing control and *orb2* RNAi WL3 brains.

Supplemental Dataset 4. Fly stocks used in this study.

Supplemental Dataset 5: Sequences of primers and smFISH probes used in this study

Supplemental Figure Legends

Figure S1: Other codon-biased reporters express at low levels in neuroblast and high levels in neurons.

A: GFP-common expresses highly throughout the brain. Scale bar is 20 µm. **B-D'**: GFP-rare^{b}, mchGFP100DV1 and GFP54C3' express highly in neurons, not in neuroblasts. **B.** GFP-rare^{b} expression in one lobe of the *Drosophila* larval brain. Scale bar is 20 µm. **C.** Schematic of codon and sequence composition of mCHGFP100DV1 reporter (Allen et al., 2022). **C'**. WL3 Larval body expression, arrows indicate testes and brain. **C"**. Expression of mCHGFP100DV1 reporter in one lobe of WL3 brain. Scale bar is 20 µm. **D.** Schematic of codon and sequence composition of

GFP54C3' reporter (Allen et al., 2022). **D'**. WL3 Larval body expression, arrows indicate testes and brain. **D"**. Expression of GFP54C3' reporter in one lobe of WL3 brain. Scale bar is 20 µm. **E.** Representative image of a MARCM clone in the central WL3 brain. Scale bar is 10 µm. Green indicates GFP-rare^b protein, magenta indicates Elav staining, gray indicates mCD8 RFP highlighting clone. Arrows indicate GFP-rare^b negative neurons. **F-I.** Representative confocal images of either GAL4 driving UAS-LacZ (**F-G)** or antibodies (**H-I**) to mark cells of interest. Scale bars are 30 µm. **F.** *OK6*-GAL4 driving UAS-LacZ expression in motor neurons in the ventral nerve cord. Some are GFP-rare^b negative (dashed outline), others are GFP-rare^b positive (solid outline). **G**. *engrailed*-GAL4 driving UAS-LacZ expression in serotonergic neurons in the ventral nerve cord. All are GFP-rare^b positive (solid outline). **H.** Wrapper antibody staining indicating midline glia in ventral nerve cord. Dashed outline indicates cell bodies without GFP-rare^b protein. **I**. Repo antibody staining in ventral nerve cord area rich in astrocyte-like glia. Most are GFP-rare^b negative, indicated by dashed outline. **J-K.** Quantification of Type I (J) and Type II (K) NB numbers in WL3 brains of control and *insc*-GAL4 driving UAS-RNAi against *brat, numb* and *prospero.* Each data point = 1 animal. Four biological replicates were performed. *=p<0.05, **=p<0.01.

****=p<0.0001 by one-way ANOVA followed by Dunnett's multiple comparisons test.

Figure S2: GFP-rare^b has abundant protein and mRNA expression specifically in neurons.

A: A WL3 GFP-rare^b brain expressing UAS-myristoylated (m) RFP driven by *asense (ase)*-GAL4 to mark NBs and GMCs and stained for mRNA molecules using smFISH. Scale bar = 10 um. **B:** A WL3 GFP-rare^b brain expressing UAS-mRFP driven by *elav*-GAL4 and stained for mRNA molecules using smFISH. Scale bar= 10 um. **C:** Quantification of GFP-rare^b protein expression

relative to w^{1118} controls in indicated cell types in WL3 brains (see Methods). Each data point = 1 animal. N = 5 animals, 2 replicates. One-way ANOVA performed followed by Tukey's multiple comparisons test, **=p<0.01. **D**. Quantification of GFP-common protein expression in indicated cell types relative to w^{1118} controls in WL3 brains. Each data point = 1 animal. N=6 animals, 2 replicates. No significant differences detected by one-way ANOVA. **E**. Relative GFP protein expression (quantitated by western blot) in 15 WL3 brains (3 replicates) expressing either GFP-rare^{b} or GFP-common, from either WT or *brat* RNAi knockdown genetic background. One way ANOVA was performed followed by Šídák's multiple comparisons test. *: p<0.05, ***p<0.005. **F**. Relative GFP mRNA, (by quantitative Real Time PCR (qRT-PCR)) in 20 brains expressing either GFP-rare^{b} or GFP-common with 3 replicates. Reporter mRNA levels are normalized to a housekeeping gene, *rp49*. Two tailed t test was performed, *: p<0.05. **G-H**. Fold change in GFP-rare^{b} protein (**G**) and mRNA (**H**) in WL3 brain cells of the indicated type, plotted the difference in the level between animals homozygous for GFP-rare^{b} and animals heterozygous for GFP-rare^{b}. At least 5 brains per condition, 2 replicates.

Figure S3: Orb2 whole-animal knockout and neuron specific knockdown result in similar decreases in GFP-rare^{b} expression. A: Knockout of *orb*2 in the entire animal leads to a more than 2-fold decrease in GFP-rare^{b} protein expression. Each data point=1 animal. N=ten animals for control, N=6 animals for *orb2Δ*, 2 replicates. Unpaired t test performed, **** = p<0.0001. **B-D**: GFP-rare^{b} protein fluorescence in WL3 brains of indicated genotypes. Scale bar = 10 µm. **E**: Quantification of GFP-rare^{b} protein fluorescence in indicated cell types in the indicated genotypes. One-way ANOVA was performed followed by Sidaks multiple comparisons test. *:

p<0.05. ****: p<0.0001. Six animals, two replicates. **F:** Quantification of GFP-common protein expression in total brain and indicated cell types for either WT (*w* RNAi) or *orb2* RNAi. Nine animals, three replicates. **G.** Quantification of the number of type I and type II neuroblasts marked by Deadpan staining in either WT (*w* RNAi) or *orb2* RNAi brains. Six animals, two replicates. **H**. Quantification of the amount of neurons in either WT (*w* RNAi) or *orb2* RNAi brains. Measured by dividing the total area of the central brain by the area covered by Elav staining. Eight animals, two replicates. **I**. Ratio of mRNA as measured by qRT-PCR for either GFP-common or GFP-rare^{b} in *orb2* knockdown brains and wild type brains. Reporter mRNA levels are first normalized to a housekeeping gene, *rp49*, and then the ratio of *orb2* knockdown to wild type is plotted. 20 animals, three replicates. Two tailed t test was performed, *: p<0.05. **J.** Quantification of GFP-common fluorescence as stained by smFISH in total brain and the indicated cell types in either *w* RNAi or *orb2* RNAi brains. Ten animals, two replicates. **K.** Quantification of GFP-rare^{b} protein expression in total brain for WT (*w* RNAi), second *CG4612* (v52947) RNAi, and second *CG13928* (v51777) RNAi compared to average fluorescence intensity of WT total brains. Each data point= 1 animal, N=10 animals, three replicates. ***=p<0.005 by Oneway ANOVA followed by Dunnett's multiple comparisons test**. L-M**: Representative confocal images of testes expressing GFP-common, scale bar is 50 µm. **L**: *vasa*-GAL4 expressing *w* RNAi. **M**: *vasa*-GAL4 expressing *orb2* RNAi. **N**: Quantification of reporter GFP-common expression in testes under either control (*w* RNAi) or *orb2* RNAi driven by *vasa*-GAL4. Line profiles of GFP-common protein expression taken from hub to spermatid (300 µm). Nine animals, three replicates. Welch's t test comparing linear regressions shows no difference.

Figure S4: **Both Orb2 binding sites and CAI are important to determine expression regulation in the *Drosophila* brain**. **A:** Graph of CAI of each codon in the sequence of the GFP-rare^{b} transgene with annotated Orb2 binding sites marked in green. **B**. Graph of CAI of each codon in the sequence of the GFP-common transgene, no Orb2 binding sites to be annotated. **C-D.** Scatter plots of number of annotated Orb2 binding sites in the 3' quartile of the CDS and the 3' UTR of transcripts of interest normalized to length, plotted against CAI of each transcript. Linear regression line plotted, r and p value for shown on the graph. **C.** Transcripts that are significantly upregulated by Orb2. **D.** Transcripts that are significantly downregulated by Orb2. **E.** Selected rare-codon-enriched genes that pass screening criteria (**Fig 5E**). Fold change values displayed in green, binding sites displayed in blue.

Figure S5: Larval expression of mGluR transgenes is responsive to codon usage changes and *orb2* expression levels, while adult expression is not. A. Western blot quantification of protein expression in the adult brain of each FLAG-tagged mGluR transgene from ten heterozygous animals, three replicates. Not significant by one-way ANOVA. **B**. Western blot quantification of protein expression in the larval brain of each FLAG-tagged mGluR transgene from 20 heterozygous animals, three replicates. One way ANOVA was performed followed by Tukey's multiple comparisons test. *: $p<0.05$, **<0.05, ***<0.005.

Figure S1: Other codon-biased reporters express at low levels in neuroblast and high levels in neurons.

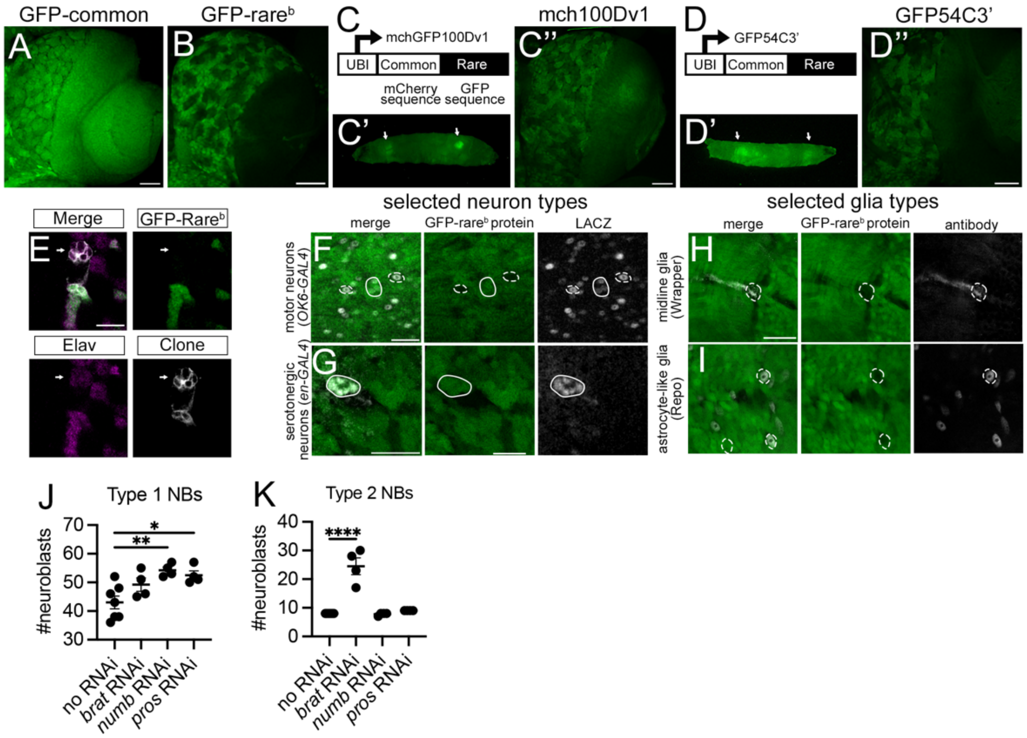

Figure S2: GFP-rare^b has abundant protein and mRNA expression in neurons, while GFP-common is expressed abundantly everywhere.

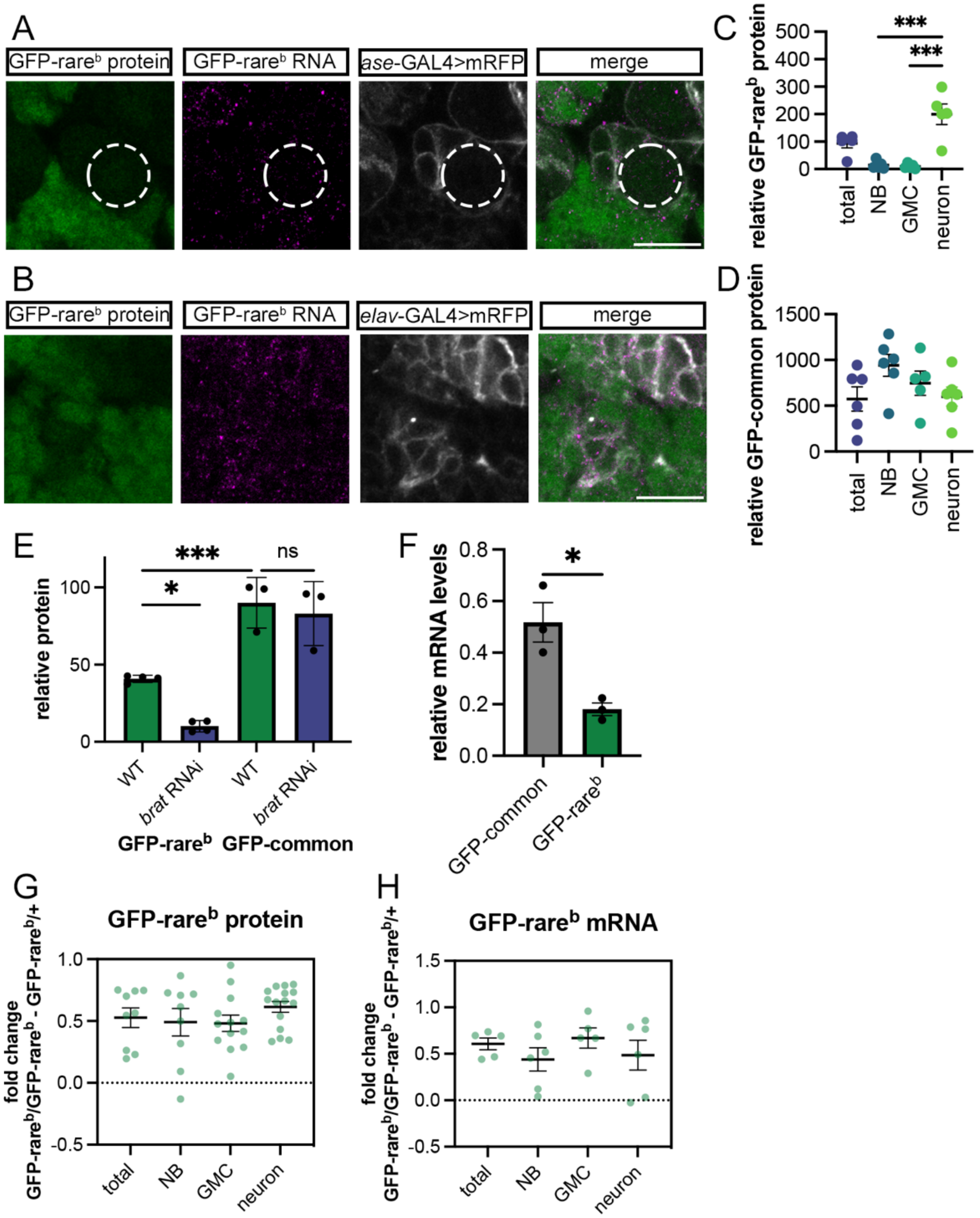

Figure S3: Orb2 whole-animal knockout and neuron specific knockdown result in similar decreases in GFP-rare^b expression

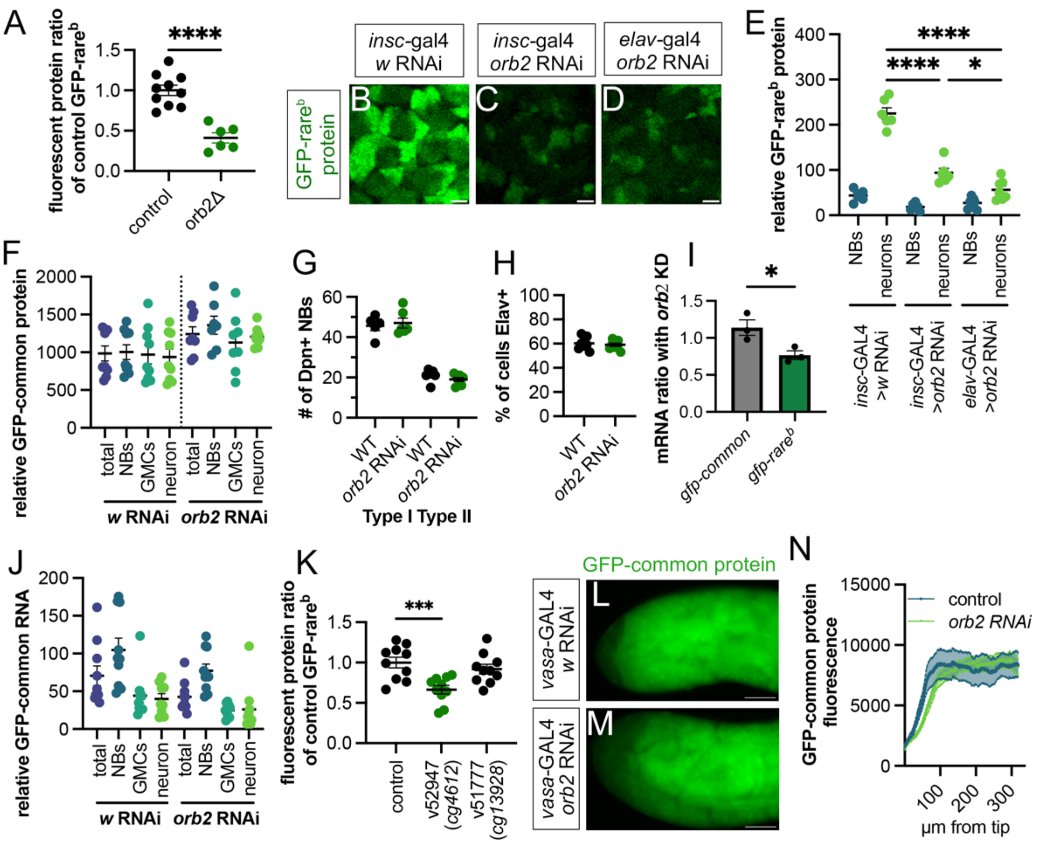

Figure S4: **Both Orb2 binding sites and CAI are important to determine expression regulation in the *Drosophila* brain**

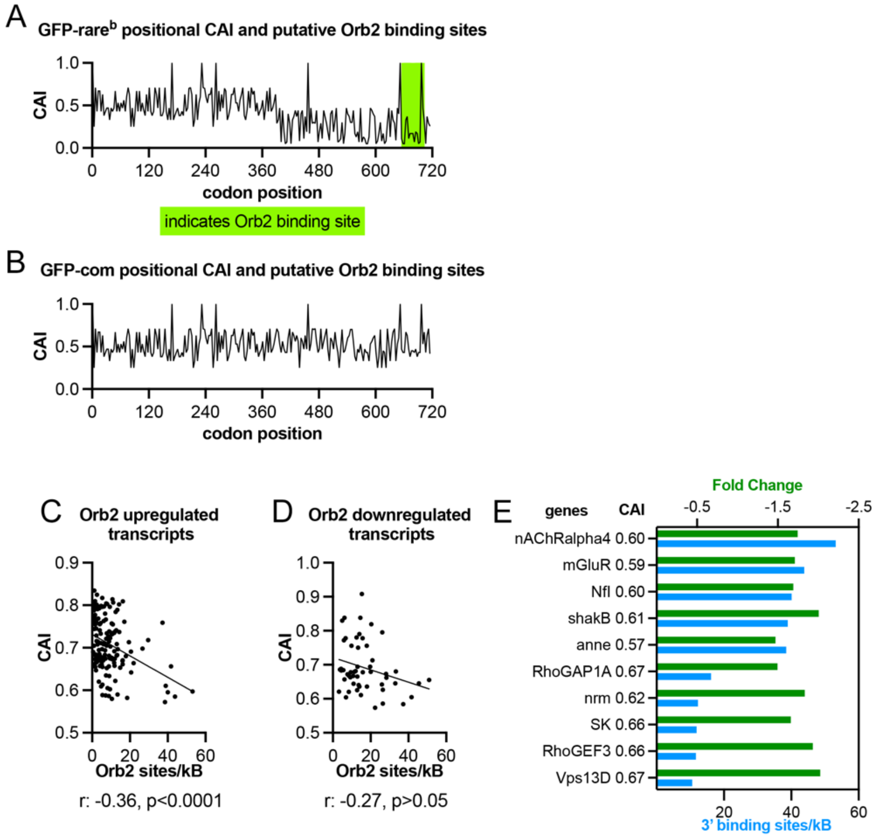

Figure S5: Larval expression of mGluR transgenes is responsive to codon usage changes and orb2 expression levels, while adult expression is not

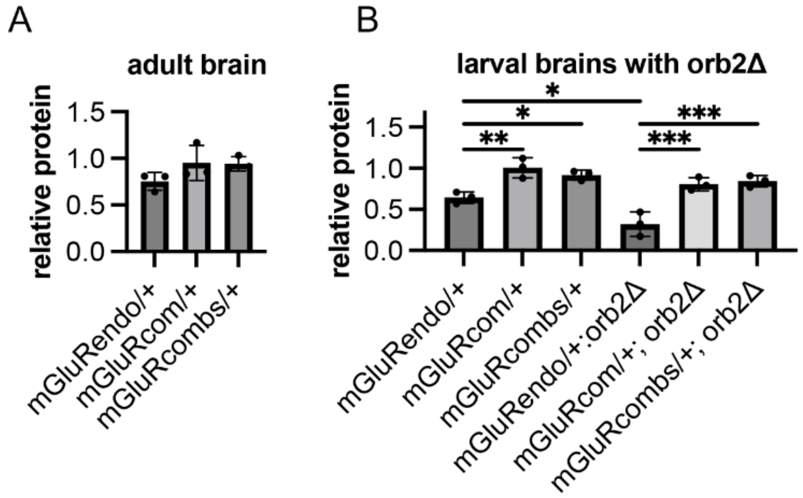

Supplement: Supplementary file 1 — Supplemental Information [file 41467_2024_48344_MOESM1_ESM.pdf]
