# Supplementary material for: Orb2 enables rare-codon-enriched mRNA expression during Drosophila neuron differentiation
Source: Nat Commun. 2024 Jun 20;15:5270. doi: 10.1038/s41467-024-48344-8 (PMC11190236; doi:10.1038/s41467-024-48344-8)
Supplement: Supplementary file 3 — Description of Additional Supplementary Information [file 41467_2024_48344_MOESM3_ESM.pdf]

Description of Additional Supplementary Information

Supplemental Dataset 1. Sequences of new transgenes presented in this study.

Supplemental Dataset 2. Data for RNAi screen.

Supplemental Dataset 3. RNAseq data comparing control and orb2 RNAi WL3 brains.

Supplemental Dataset 4. Fly stocks used in this study.

Supplemental Dataset 5. Sequences of primers and smFISH probes used in this study
