# Supplementary material for: Orb2 enables rare-codon-enriched mRNA expression during Drosophila neuron differentiation
Source: Nat Commun. 2024 Jun 20;15:5270. doi: 10.1038/s41467-024-48344-8 (PMC11190236; doi:10.1038/s41467-024-48344-8)
Supplement: Supplementary file 9 — Reporting Summary [file 41467_2024_48344_MOESM9_ESM.pdf]

nature portfolio

Corresponding author(s): Don Fox

Last updated by author(s): 2024-04-09

Reporting Summary

Nature Portfolio wishes to improve the reproducibility of the work that we publish. This form provides structure for consistency and transparency in reporting. For further information on Nature Portfolio policies, see our Editorial Policies and the Editorial Policy Checklist.

Statistics

For all statistical analyses, confirm that the following items are present in the figure legend, table legend, main text, or Methods section.

n/a	Confirmed	
☐	☒	The exact sample size (*n*) for each experimental group/condition, given as a discrete number and unit of measurement
☐	☒	A statement on whether measurements were taken from distinct samples or whether the same sample was measured repeatedly
☐	☒	The statistical test(s) used AND whether they are one- or two-sided<br>*Only common tests should be described solely by name; describe more complex techniques in the Methods section.*
☐	☒	A description of all covariates tested
☐	☒	A description of any assumptions or corrections, such as tests of normality and adjustment for multiple comparisons
☐	☒	A full description of the statistical parameters including central tendency (e.g. means) or other basic estimates (e.g. regression coefficient) AND variation (e.g. standard deviation) or associated estimates of uncertainty (e.g. confidence intervals)
☐	☒	For null hypothesis testing, the test statistic (e.g. F, t, r) with confidence intervals, effect sizes, degrees of freedom and P value noted<br>*Give P values as exact values whenever suitable.*
☒	☐	For Bayesian analysis, information on the choice of priors and Markov chain Monte Carlo settings
☒	☐	For hierarchical and complex designs, identification of the appropriate level for tests and full reporting of outcomes
☐	☒	Estimates of effect sizes (e.g. Cohen's d, Pearson's r), indicating how they were calculated

Our web collection on statistics for biologists contains articles on many of the points above.

Software and code

Policy information about availability of computer code

Data collection	Nikon NIS Elements was used to acquire images. Western blotting data was acquired using a LICOR Odyssey CLX.
Data analysis	Image J was used for Image analysis; Graph Pad Prism was used for statistical analysis.<br>Bulk RNA-sequencing: Raw reads were mapped to D. melanogaster genome (BDGP6) using HISAT2 and counts assigned to protein-coding genes were calculated using featureCounts (v1.6.3). DESeq2 was then used to identify significantly differently expressed genes using the following parameters: Padj<0.01, and the absolute value of log2 FC>0.5.

For manuscripts utilizing custom algorithms or software that are central to the research but not yet described in published literature, software must be made available to editors and reviewers. We strongly encourage code deposition in a community repository (e.g. GitHub). See the Nature Portfolio guidelines for submitting code & software for further information.

Data

Policy information about availability of data

All manuscripts must include a data availability statement. This statement should provide the following information, where applicable:

- Accession codes, unique identifiers, or web links for publicly available datasets
- A description of any restrictions on data availability
- For clinical datasets or third party data, please ensure that the statement adheres to our policy

No restrictions on data availability for any of these datasets used in the manuscript. RNA sequencing published in this manuscript: GSE263513. Previously published GEO datasets used: GSE71104, GSE38764, GSE59611

Research involving human participants, their data, or biological material

Policy information about studies with human participants or human data. See also policy information about sex, gender (identity/presentation), and sexual orientation and race, ethnicity and racism.

Reporting on sex and gender	N/A
Reporting on race, ethnicity, or other socially relevant groupings	N/A
Population characteristics	N/A
Recruitment	N/A
Ethics oversight	N/A

Note that full information on the approval of the study protocol must also be provided in the manuscript.

Field-specific reporting

Please select the one below that is the best fit for your research. If you are not sure, read the appropriate sections before making your selection.

☒ Life sciences ☐ Behavioural & social sciences ☐ Ecological, evolutionary & environmental sciences

For a reference copy of the document with all sections, see nature.com/documents/nr-reporting-summary-flat.pdf

Life sciences study design

All studies must disclose on these points even when the disclosure is negative.

Sample size	Sample sizes were determined based on variations in genotypes and treatments.
Data exclusions	No data were excluded from the analysis
Replication	Multiple independent RNAi lines were used. All experiments were reproduced 2-3 times and all data used.
Randomization	animals were allocated based on genotype
Blinding	given that we used randomization and replication, along with quantitative measures, we did not also include blinding

Reporting for specific materials, systems and methods

We require information from authors about some types of materials, experimental systems and methods used in many studies. Here, indicate whether each material, system or method listed is relevant to your study. If you are not sure if a list item applies to your research, read the appropriate section before selecting a response.

Materials & experimental systems

n/a	Involved in the study	
☐	☒	Antibodies
☒	☐	Eukaryotic cell lines
☒	☐	Palaeontology and archaeology
☐	☒	Animals and other organisms
☒	☐	Clinical data
☒	☐	Dual use research of concern
☒	☐	Plants

Methods

n/a	Involved in the study	
☒	☐	ChIP-seq
☒	☐	Flow cytometry
☒	☐	MRI-based neuroimaging

Antibodies

Antibodies used	Primary antibodies included Dpn (1:250, Abcam ab195173), Hts (1:200, Developmental Studies Hybridoma Bank, DSHB1b1) Elav (1:500, DSHB 7E8A10), Wrapper (1:100, DSHB 10D3), Repo (1:500, DSHB 8D12), FLAG M2 (1:500, Sigma, F3165), α-tubulin (1:2,000, Sigma, ABT170), GFP (1:1000, Life Technologies, #A11122). Secondary antibodies : Goat anti-rat or mouse (1:5000 for IF; goat-anti-rat IgG (H+L) Alexa Fluor 647, Invitrogen A-21247, goat-anti-mouse IgG (H+L) Alexa Fluor 647, Invitrogen A-21235), Donkey Anti rabbit or mouse 1:10000 (IRDye 800CW IgG Secondary Antibody (clonality unspecified) LICOR 926–32213)
Validation	All antibodies used in this work are commercially available and the validation can be accessed at manufactures website.

Animals and other research organisms

Policy information about studies involving animals; ARRIVE guidelines recommended for reporting animal research, and Sex and Gender in Research

Laboratory animals	Drosophila melanogaster - larvae and adults
Wild animals	none
Reporting on sex	n/a
Field-collected samples	No field collected samples were used in this study
Ethics oversight	No ethical approval required

Note that full information on the approval of the study protocol must also be provided in the manuscript.

Plants

Seed stocks	*Report on the source of all seed stocks or other plant material used. If applicable, state the seed stock centre and catalogue number. If plant specimens were collected from the field, describe the collection location, date and sampling procedures.*
Novel plant genotypes	*Describe the methods by which all novel plant genotypes were produced. This includes those generated by transgenic approaches, gene editing, chemical/radiation-based mutagenesis and hybridization. For transgenic lines, describe the transformation method, the number of independent lines analyzed and the generation upon which experiments were performed. For gene-edited lines, describe the editor used, the endogenous sequence targeted for editing, the targeting guide RNA sequence (if applicable) and how the editor was applied.*
Authentication	*Describe any authentication procedures for each seed stock used or novel genotype generated. Describe any experiments used to assess the effect of a mutation and, where applicable, how potential secondary effects (e.g. second site T-DNA insertions, mosiacism, off-target gene editing) were examined.*
